# Covert Communication through Robust Fragment Hiding in a Large Number of Images

**DOI:** 10.3390/s24020627

**Published:** 2024-01-18

**Authors:** Pengfei Wang, Hua Zhong, Yapei Feng, Liangbiao Gong, Yuxiang Tang, Zhe-Ming Lu, Lixin Wang

**Affiliations:** 1 School of Communication Engineering, Hangzhou Dianzi University, Hangzhou 310018, China; pfwanghdu@163.com (P.W.); wanglixin@hdu.edu.cn (L.W.); 2 School of Cyberspace, Hangzhou Dianzi University, Hangzhou 310018, China; fengyapei@hdu.edu.cn (Y.F.); gongliangbiao@hdu.edu.cn (L.G.); tyx09122023@163.com (Y.T.); 3 School of Aeronautics and Astronautics, Zhejiang University, Hangzhou 310027, China; 4 Center for Generic Aerospace Technology, Huanjiang Lab, Zhuji 311816, China

**Keywords:** robust image watermarking, covert communication, redundant fragment hiding, steganography

## Abstract

For covert communication in lossy channels, it is necessary to consider that the carrier of the hidden watermark will undergo multiple image-processing attacks. In order to ensure that secret information can be extracted without distortion from the watermarked images that have undergone attacks, in this paper, we design a novel fragmented secure communication system. The sender will fragment the secret data to be transmitted and redundantly hide it in a large number of multimodal carriers of messenger accounts on multiple social platforms. The receiver receives enough covert carriers, extracts each fragment, and concatenates the transmitted secret data. This article uses the image carrier as an example to fragment the text file intended for transmission and embeds it into a large number of images, with each fragment being redundant and embedded into multiple images. In this way, at the receiving end, only enough stego images need to be received to extract the information in each image, and then concatenate the final secret file. In order to resist various possible attacks during image transmission, we propose a strong robust image watermarking method. This method adopts a watermark layer based on DFT, which has high embedding and detection efficiency and good invisibility. Secondly, a watermark layer based on DCT is adopted, which can resist translation attacks, JPEG attacks, and other common attacks. Experiments have shown that our watermarking method is very fast; both the embedding time and the extraction time are less than 0.15 s for images not larger than 2000×2000. Our watermarking method has very good invisibility with 41 dB PSNR on average. And our watermarking method is more robust than existing schemes and robust to nearly all kinds of attacks. Based on this strong robust image watermarking method, the scheme of fragmenting and hiding redundant transmission content into a large number of images is effective and practical. Our scheme can 100% restore the secret file completely under different RST or hybrid attacks, such as rotation by 1 degree and 5 degrees, scaling by 1.25 and 0.8, and cropping by 10% and 25%. Our scheme can successfully restore the secret file completely even if 30% of received images are lost. When 80% of received images are lost, our scheme can still restore 61.1% of the secret file. If all stego images can be obtained, the original text file can be completely restored.

## 1. Introduction

In the modern world, the importance of protecting digital data is very high. Information security is an important part of cyberspace security, ensuring that secret data are not illegally stolen, altered, or damaged by malicious individuals during the transmission phase. It is not only a national matter, but also a matter related to the interests of the people, and is related to a harmonious society. One of the methods to ensure the security of digital data is to use steganography. This is particularly applicable to various multimedia data, such as images, audio, videos, etc. The use of steganography to hide information is an increasingly mature and developing scientific field.

In order to clarify the technical background more clearly, we strictly distinguish the concepts of steganography and watermarking here. Steganography schemes mainly focus on capacity, invisibility, and security, and their protected object is confidential information; watermarking schemes mainly focus on robustness and invisibility, and their protected object is the carrier. Current steganography communication mostly adopts steganography technology, seldom considering the use of robust watermarking technology in noisy channels under big data backgrounds. This article mainly focuses on image carriers. Therefore, we first introduce the current state of image steganography, and then put forward the idea of introducing robust image watermarking schemes for secure communication in noisy channels, grounded in a big data context. Thus, we analyze the current situation of existing robust image watermarking schemes, and then address their shortcomings, and finally introduce our robust image watermarking method and the fragmented covert communication scheme based on this method.

The technical idea of image steganography is to invisibly modulate secret information into the carrier image and transmit it through open channels. This not only hides the transmitted information but also conceals the existence of secret communication, making it a safe and reliable information transmission method in network environments. In particular, if the image steganography scheme can adapt to open network channels, it can more effectively achieve convenient and secure covert communication of secret information. Traditional image steganography techniques are usually based on the assumption of lossless channels [1]. In the past ten years, many traditional image steganography schemes have been proposed. They can be mainly divided into seven categories [2]: (1) LSB (least significant bit)-based scheme [3]. The method [3] has very good invisibility but insufficient capacity. It avoids the overflow and underflow problem, but steganalysis is missing; therefore, it is hard to predict the dependability of the work. (2) LSB matching-based scheme [4]. The method [4] has outstanding invisibility but unsatisfactory capacity. It has good resistance against regular and singular (RS) analysis and pixel difference histogram (PDH) analysis. (3) PVD (pixel value differencing)-based scheme [5]. The method [5] has very good invisibility but unsatisfactory capacity. It considers robust techniques as multi-directions, but it is unacceptable for approaches needing supplementary capacity. (4) PVD+LSB-based scheme [6]. The method [6] has outstanding invisibility and high capacity. However, steganalysis is missing; therefore, it is hard to predict the dependability of the work. (5) MF (modulus function)+PVD-based scheme [7]. The method [7] has very good invisibility and satisfactory capacity, and it avoids the falling-out boundary issue. However, PDH analysis is missing; therefore, it is hard to predict the dependability of the work. (6) RDH (reversible data hiding) + MF-based scheme [8]. The method [8] has satisfactory invisibility and satisfactory capacity, and it withstands histogram analysis. However, its capacity and invisibility can be further improved, therefore, it is hard to predict the dependability of the work. (7) RDH+PVO (pixel value ordering)-based scheme [9]. The method [9] has outstanding invisibility but unsatisfactory capacity. It has better results compared to existing RDH+PVD techniques. However, steganalysis is missing; therefore, it is hard to predict the dependability of the work.

**Figure 1:**
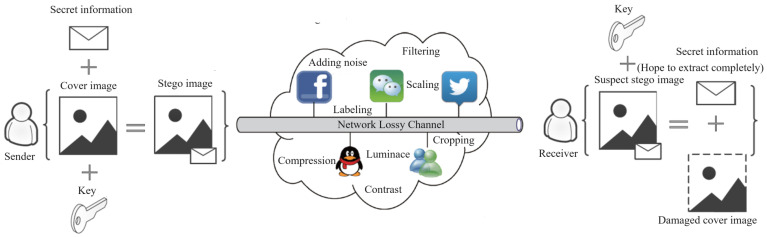
Schematic diagram of open network channel image steganography communication.

The main problem with traditional image steganography schemes is that they lean toward capacity but give insufficient attention to robustness. However, in actual open network channels, it is not only necessary for the stego image, produced after steganography, to be indistinguishable from the original carrier image to the naked eye, but also essential to ensure that the embedded secret information can be correctly extracted after being subjected to various image-processing methods or attacks that may exist in the channel. As shown in Figure 1, after the sender embeds secret information in the carrier image, the stego image output is often transmitted over open network channels, e.g., social platforms such as QQ, Facebook, Twitter, and WeChat. Due to bandwidth limitations and limited processing power, uploaded stego images must undergo lossy operations such as compression and scaling, resulting in a certain degree of image degradation [10]. After receiving the stego image, which may have been subjected to attack processing, in order to extract the secret information completely and reliably, it is necessary to use strong robust embedding and extraction algorithms in algorithm design.

In response to the aforementioned practical needs, many scholars have achieved fruitful research results in robust image steganography after years of research and exploration efforts. Robust steganography technology focuses on solving the invisibility and extraction accuracy of the embedded secret information in the stego image after various processing attacks. The basic idea is to comprehensively consider the anti-attack ability and imperceptibility during embedding, and combine the embedding cost minimization coding method to achieve high reliability and large embedding capacity for secure communication. For the demand of covert communication in lossy network channels, the commonly used methods for robust image steganography schemes include: (1) constructing or finding specific carriers with strong resistance to attacks, usually robust transform domain coefficients [11], invariant features, and robust relative relationships; (2) estimating embedding losses and costs [12], and comprehensively examining attack resistance and imperceptibility; (3) finding the optimal embedding region, which generally requires the region to be embedded to have complex textures and be difficult to model statistically [13]; (4) integrating source/channel encoding technology with the idea of minimizing embedding distortion encoding [14]; (5) combining stable, reliable, and covert security strategies [15]. The purpose of these five technical measures is to design information embedding and extraction methods that are sufficiently robust to common processing operations such as compression and scaling. These methods can make up for the shortcomings of previous traditional image steganography algorithms in terms of both attack resistance and invisibility, thereby becoming a new focus of attention in the field of image information hiding.

The architecture of robust image steganography technology [11,12,13,14,15] includes six parts, namely: how to filter carrier images, how to find or construct robust carriers, what embedding cost measures to use, how to filter embedding regions, how to combine source/channel encoding, and how to design security policies. The first step is to screen suitable carrier images. Not all images are suitable as covert communication carriers, and it is usually necessary to select images with complex textures that make it difficult to see the embedded information after hiding it as the carrier. The second step is to find or construct a robust vector. The purpose is to design transform domain coefficients or features or relative relationships that are stable and robust to various image-processing attacks while balancing invisibility and robustness. The third step is to choose a reasonable embedding cost measure. The core purpose is to measure the embedding cost of carrier elements, fully consider robustness and invisibility, and provide quantitative indicators. The fourth step is to filter the embedded region or channel. The core purpose is to fully consider the characteristics of lossy channels to select image regions with complex textures and robust features to hide information, leaving enough modification space to optimize performance. The fifth step is to combine source/channel encoding. Its core purpose is to design effective error control coding methods and provide a theoretical basis for secure and reliable transmission. The sixth step is to design a security strategy. Its purpose is to design communication protocols and strategies, as well as provide application guidance. From the above six aspects, it can be seen that robust image steganography technology needs to have strong security, like adaptive image steganography technology, and strong robustness, like robust image watermarking technology. It also needs to consider both the detection resistance of disguised images and the robustness of embedded information.

In this paper, we consider the situation where we have big image databases in hand and we need to transmit a secret file over social platforms with attacks. An immediate idea involves fragmenting the secret file and hiding the fragments within a number of images using robust image steganography schemes. In fact, any robust watermarking method can be also adopted in this fragmented information-hiding situation based on big image databases, since in this situation, the requirements of steganalysis security and capacity for a single image are largely weakened. That is, under the big data situation, capacity and security are not so important for a single image, while the robustness against attacks during the transmission is very important. Then, it is proposed that robust image watermarking technology can be fully utilized to solve the problem in noisy channels under big data environments. Robust watermarking technology aims to solve the problem of accurately detecting watermark information even when watermarked images are subjected to various attacks and processing. The basic idea is to construct an embedding domain that is sufficiently robust to various common attacks [16]. It also requires the fusion of redundant embedding and error correction coding schemes to achieve efficient and robust information embedding and extraction. Robust image watermarking algorithms include spatial domain-based methods, transform domain-based methods, and other methods. Currently, the main research field regarding image watermarking focuses on transform domain-based methods.

The spatial domain-based image watermarking algorithm embeds watermarks by modifying the pixel values of the host image. Old algorithms, including LSB replacement algorithms, will not be mentioned here. The latest algorithms are as follows: Ghadi et al. [17] proposed a spatial domain image watermarking method, which utilizes association rule mining and texture analysis of digital images to embed watermarks in strong texture regions. This not only achieves good imperceptibility but also enhances the ability to resist attacks. Kumar and Singh [18] proposed a spatial domain image watermarking method that combines the Hill cipher and LSB (least significant bit) substitution. The two-dimensional code is embedded into the grayscale host image, which has good invisibility but general robustness. Liu et al. [19] proposed a watermarking scheme based on the logistic mapping scrambling algorithm and RSA asymmetric encryption algorithm to provide security for data with large embedding capacity. In addition, reference [20] proposed a moment-based watermarking method.

Frequency domain watermarking first involves transforming the host image, commonly including matrix decomposition, three major transformations (namely discrete wavelet transform (DWT) [21,22,23,24,25], discrete cosine transform (DCT) [26,27,28,29,30], and discrete Fourier transform (DFT) [31,32,33,34]), and other transformations [20,35,36,37,38,39], followed by modifying the transform coefficients to embed the watermark.

Guo et al. [21] proposed a new method for DWT (discrete wavelet transform), i.e., QRD (QR decomposition) subspace image watermarking based on the FA (firefly algorithm). Reference [22] proposed an image watermarking scheme in the wavelet domain with optimized compensation of a singular value decomposition via the artificial bee colony. In [23], a hybrid watermarking scheme based on DWT and SVD in addition to a deep belief neural (DBN) network was proposed. However, this method does not perform well for image-processing attacks with severe parameters. Amini et al. [24] proposed a wavelet-based watermark decoder based on vector HMM (the hidden Markov model) because they can capture the distribution of subband edges and fully incorporate the cross-scale and cross-directional dependencies of wavelet coefficients. In [25], an image watermarking scheme by using advantages of both frequency domain and wavelet domain was proposed, which is very robust to Gaussian noise, but the robustness against geometrics is not high and the complexity is not low.

The algorithm based on two-dimensional discrete cosine transform (2D-DCT) proposed by Yuan et al. [26] can effectively resist multiple attacks. Earnawan and Kabir [27] proposed a watermarking technique based on the optimal DCT psychological visual threshold, which embeds scrambled watermark bits into certain frequency regions of the DCT to minimize the impact of image distortion. Zhou et al. [28] proposed a GCC-based watermarking method to improve robustness against geometric transformation attacks. Zong et al. [29] proposed a level-based watermarking method, which uses 2D-DCT on each host image and embeds the watermark into the DCT coefficients of the host image block using rank-based embedding rules. The watermark is extracted by comparing the levels of the detection matrix generated from the received image using a key, achieving a very high embedding capacity. The scheme proposed by Hatoum et al. [30] adopts spread transform dithering modulation (STDM), which applies DCT to the host image when embedding the watermark, and then embeds the watermark bit through NSTDM (normalized STDM). After applying IDCT in the extraction stage, the NSTDM decoder is implemented to detect the watermark bit.

Jamal et al. [31] proposed a watermarking algorithm based on replacement boxes and DFT; it is highly robust and secure against different types of attacks, but also has high complexity. Liao and Yin [32] proposed a watermarking algorithm using DFT and coefficient classification, which has good imperceptibility and robustness, but takes a long time. Reference [33] proposed a perceptual DFT watermarking scheme with improved detection and robustness to geometrical distortions. In 2021, Begum and Uddin [34] implemented a secure and robust DFT-based image watermarking scheme through hybridization with the decomposition algorithm.

Reference [35] achieved watermark embedding and extraction by discovering and utilizing the correlation between the two coefficients in the U matrix of SVD. Liu et al. [36] presented a fusion-domain color image watermarking scheme based on Haar transform and image correction. Wang et al. [37] proposed a blind watermarking algorithm for dual-color images using discrete Hartley transform (DHT). It mainly used the image’s geometric features, such as sides and angles, to correct the attacked image and embedded a color watermark into a color image with a large embedding capacity and strong practicability. However, this algorithm is not robust to rotation of 90°. The color image watermarking using new fractional-order exponent moments in [20] is very robust to different kinds of geometric distortions and attacks, but the complexity is very high and the efficiency is not high enough for industrial use. In [38], a blind watermarking algorithm based on contourlet transform with singular value decomposition was proposed, but this method is not robust enough to rotation and collage attacks and the complexity is high. In [39], synchronization correction is used in robust image watermarking.

There are also some reversible watermarking schemes. Meng et al. [40] proposed a reversible watermark based on integer wavelet transform (IWT) that embeds binary watermarks into the diagonal components of the grayscale main image. Its invisibility is very good, but the embedding ability and robustness need to be improved. Wang et al. [41] proposed a scheme for implementing reversible watermarks using prediction error extension (PEE), which fully combines the advantages of PEE and histogram shift, which can improve prediction accuracy and achieve good results. In [42], a robust and reversible color image watermarking algorithm in the spatial domain fusing discrete Fourier transform (DFT) was proposed, but the scheme is not robust to rotation and collage, and the complexity is not low. In addition to digital image hiding, there are also some other security schemes for images, such as visual cryptography (VC) schemes [43,44]. In VC, from one individual visual key, no information about the secret image can be extracted. After the multiple visual keys are overlapped, the secret image can be displayed but each individual key cannot be directly observed from the overlapped result. This is another solution to hiding secret information.

Although the above robust image watermarking schemes can be applied to the fragmented information-hiding situation, many recent robust watermarking schemes are not fast enough and are not so robust to combined attacks, especially combined RST attacks. In fact, although image watermarking techniques can be used in many scenarios, they have not been large-scale used yet in the industry. There are two reasons, one is that the watermarking efficiency is often ignored; the other is that the scheme cannot resist various attacks. From above, we can see that many existing state-of-the-art methods based on different domains cannot achieve both high robustness and fast speed and, thus, they are not so suitable to industrial circles. In order to apply digital watermarking techniques to covert communication with robust performance, we provide a multi-domain image watermarking scheme, including DFT-based watermarking and DCT-based watermarking layers. The first watermarking layer is based on DFT for image synchronization, and the second watermarking layer is based on DCT for embedding information.

The rest of this paper is organized as follows. In Section 2, a detailed scheme, including the hiding and extracting processes, is described. Section 3 is the experimental part with analysis. Finally, the conclusions will be shown in Section 4.

## 2. Proposed Scheme

### 2.1. Basic Idea

Although traditional steganography schemes offer good security, they cannot be directly applied to real social network platforms. Due to limited memory and bandwidth, stego images uploaded to social networking platforms inevitably undergo various lossy operations, such as reapplying jpeg compression to reduce size. However, traditional steganography schemes mainly considered an ideal laboratory environment, resulting in most of these schemes being unsuitable for real communication network platforms. Nowadays, almost everyone possesses a smartphone and is accustomed to using social network platforms to share music, images, and videos, which has created a covert communication ecosystem. Therefore, there is significant potential to develop robust covert communication schemes based on robust steganography/watermarking methods for social network platforms.

In this paper, we propose a novel fragmented secure communication system for covert communication in lossy channels, fully considering various attacks that images may experience in these channels. The main purpose of this system is to achieve fragmented steganographic communication across multiple shards and social platforms. The sender fragments the secret data to be transmitted and redundantly hides it in a large number of multimodal carriers of messenger accounts on multiple social platforms. The receiver, after receiving enough covert carriers, extracts each fragment and concatenates the transmitted secret data. This article, taking image carriers as an example, fragments the text files to be transmitted and embeds them into a large number of images. One fragment needs to be redundantly embedded into multiple images. Thus, at the receiver, only enough stego images need to be received to extract the information in each image, and then concatenate the final secret file.

In the following subsections of this section, we first introduce the novelty of the proposed scheme. We then introduce the core watermarking scheme used in the covert communication framework. Finally, we detail our secret hiding process and secret extraction process using the core watermarking scheme based on a large image database.

### 2.2. The Novelty of the Proposed Scheme

In this subsection, we would like to emphasize the novelty of our scheme. We describe our novelty in terms of two aspects. One is related to the whole framework. The other is related to our core watermarking method.

#### 2.2.1. The Novelty of the Whole Framework

The novelty of the proposed covert communication framework lies in three aspects:

First, we propose the novel idea of splitting the secret file into fragments, and then embedding each fragment in an image. Here, one fragment is redundantly embedded into several images. Thus the whole secret file can be redundantly hidden in a big image database.

Second, we embed both the fragment number and the fragment content into an image. Thus, the receiver can extract the fragment number and its related content from each stego image. Thus, the receiver can restore the secret file as long as it gathers enough stego images.

Third, we adopt our own robust watermarking method in the proposed covert communication framework. Our watermarking method is very robust against nearly all kinds of common attacks, which cover the operations that most of the social platforms will perform during transmission.

#### 2.2.2. The Novelty of Our Watermarking Method

In our covert communication framework, based on social platforms with big data, a novel watermarking method is designed. It seems that our method is just a multi-domain-based method. In fact, there are some special points, unlike other existing methods, that make our watermarking method very robust to nearly all kinds of attacks.

First, a DFT-based watermarking layer is proposed for synchronization with high embedding and detection efficiency; this method also has good imperceptibility. To save the computation time, we convert the DFT-based transform domain method into a spatial domain-based method with better imperceptibility and robustness. The new principles for constructing the 0,1-sequence are given. The template is prepared in advance to greatly reduce the required embedding time. The required detection time is reduced by cropping the image and using the polar transformation and filter. We also design a new method for determining the rotation angle and scale factor during extraction. Thus our method is therefore faster than many existing DFT-based watermarking schemes.

Second, a DCT-based watermarking layer is proposed for resisting translation attacks, JPEG attacks, and other common attacks. On the one hand, we use fast algorithms to calculate DCT. On the other hand, we use the information header to resist the translation attack and shear attack. Thus, our embedding scheme is both efficient and robust.

Third, the watermarking scheme integrates many techniques because it is designed for industrial use with high efficiency, good robustness, and good imperceptibility. We combine several techniques, such as synchronization correction, information header, and error-correcting code—during both the embedding and extraction processes—to make our scheme fast enough as well as robust to most attacks that might in real applications. Thus, our method is superior to existing methods since they use fewer techniques in their scheme and do not think of the real industrial application.

### 2.3. Proposed Robust Watermarking Algorithm

To implement our fragmented secure communication system for covert communication scenarios in lossy channels, we need to adopt a strongly robust image watermarking algorithm as the core watermarking algorithm of this system. Thus, we designed a specific robust watermarking scheme for our system. The detailed embedding and extraction methods are described as follows:

#### 2.3.1. Proposed Two-Stage Embedding Algorithm

Our core embedding algorithm includes two stages. The first embedding stage is based on DFT for image synchronization. The second embedding stage is based on DCT for embedding information.

(1) DFT-based watermark embedding stage.

DFT is a suitable transform that can be used for synchronization correction. If the image is rotated or scaled, the 2D DFT spectrum of the image will rotate or scale as well. Therefore, by using these features, we can obtain the scaling rate and the rotation angle of the image with a high accuracy during extraction.

**Figure 2:**
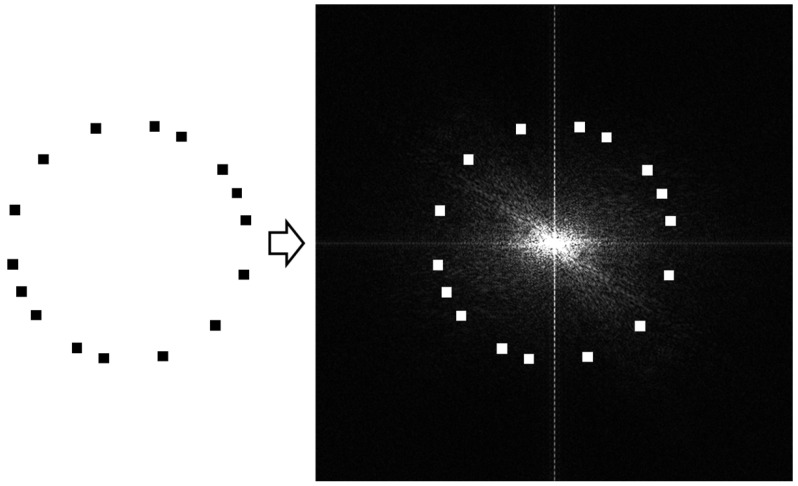
A DFT-based embedding example.

The implementation process of this method can be concisely described as follows: First, the image should be a square. If the image is not a square, then it should be padded to be a square. Second, DFT is performed on the image to obtain the 2D spectrum. Third, a pseudo-random {0,1}-sequence is generated and the spectral amplitude is changed according to the sequence. The amplitudes to be changed are located on a circle. If the bit in the sequence is 0, the amplitude is unchanged; if the bit in the sequence is 1, the amplitude is enhanced. An example can be shown in Figure 2. Finally, IDFT is performed on the spectrum to obtain the watermarked image. Thus, in the extraction process, by computing the cross-covariance value between the embedded pseudo-random sequence and the circle spectral amplitude sequence of the input image, the rotation angle and scaling factor can be obtained.

The above method can obtain good results, but the embedding process takes much time, the embedding strength is not flexible, and the imperceptibility is not good. In addition, the extraction process requires exhaustive search, which has low efficiency. In order to deal with these problems, we designed a new DFT-based method for synchronization.

The proposed method is similar to the above method in principle, but the implementation is different. In our method, before the embedding, a spatial domain template should be constructed. The spatial domain template is a residual result, and the construction process can be summarized as follows:

(1) template=α(IDFT(EMBED(DFT(G)))−G)

**Figure 3:**
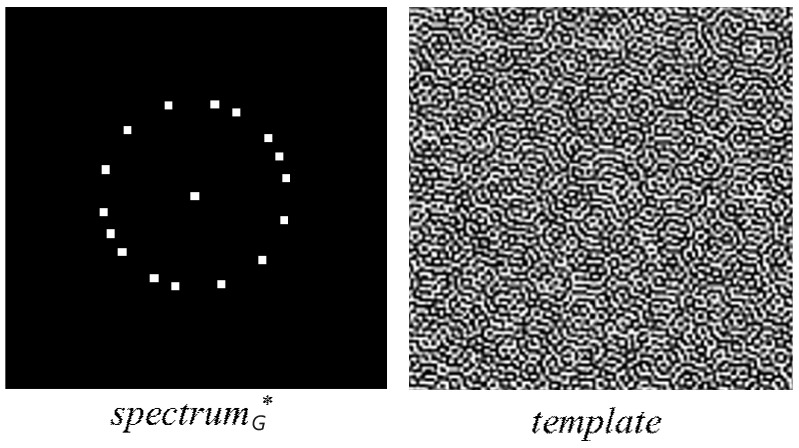
A template construction example.

In Equation (1), α indicates the weight parameter, *G* denotes a grayscale image with a large square size. Here, all pixel values in image *G* are the same. To avoid numerical overflow, we usually choose 128 as the same pixel value. In Equation (1), the “EMBED” function enhances the corresponding amplitudes of $spectrum_{G}=DFT\left(G\right)$ to be $spectrum_{G}^{*}=EMBED\left(DFT\left(G\right)\right)$ based on the traditional method, but the 0,1-sequence rather than the pseudo-random sequence is embedded. The principles of constructing the 0,1-sequence are as follows: (1) the sequence should not be periodic; (2) the sequence should be asymmetric; (3) the total number of 0 s and 1 s in the sequence should be balanced. A template construction example is shown in Figure 3.

The template-embedding process can be described as the following equation:

(2) $$I^{w}=I+template_{crop}\times gain$$

where *I* denotes the carrier image, $I^{w}$ denotes the embedded image, and $template_{crop}$ denotes the cropped template from the center of the template in Equation (1). Note that $template_{crop}$ and the carrier image have the same size. And gain denotes the adaptive gain designed for controlling the local weight of the template. Generally, smooth areas are weighted lower, and rough areas are weighted higher.

The proposed DFT-based watermarking method is robust, but it is not easy to embed enough information. Therefore, the DCT-based watermarking method is proposed to embed the customized information.

(2) DCT-based watermark embedding stage

**Figure 4:**
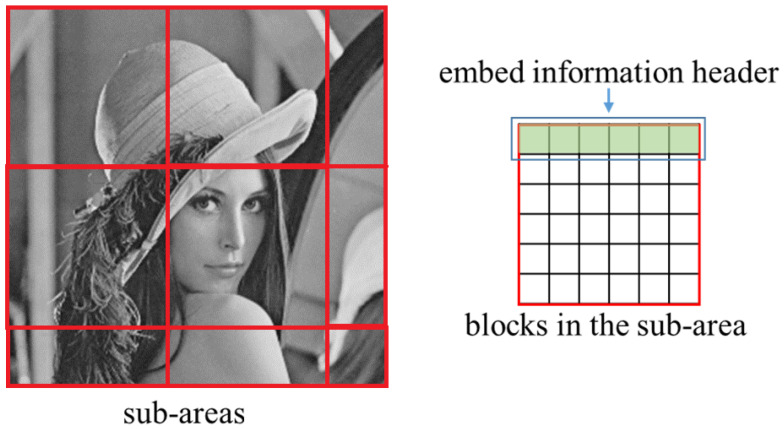
An example of sub-areas and blocks in the sub-area. The position for embedding information header in the sub-area is shown as well.

For the DCT-based watermarking stage, the embedding process is simple. The image after DFT embedding is input and divided into blocks, and then DCT is performed on each block. The information is embedded by modifying DCT coefficients. Before modification, the independent sub-area should be settled in the image, as shown in Figure 4. In each independent sub-area, the encoded information will be embedded once. Additionally, the information header should be embedded in each sub-area to resist the translation attack, where the header information is embedded into the blocks in the first row. Then, a pair of DCT coefficients of the block is chosen, the difference between the two coefficients is computed, and then the QIM (quantization index modulation) is used to modify the difference for embedding the information.

In the practical applications, some techniques or tips can be used to improve the performance: first, to compile the program faster, with the same algorithm, C++ is more efficient than Python; second, float numbers can be replaced by integer numbers for higher efficiency; third, the information to be embedded needs to be encoded before the embedding; finally, encryption or scrambling can enhance the security, and error-correcting code can be used for error correction.

(3) The whole two-stage watermark embedding process.

**Figure 5:**
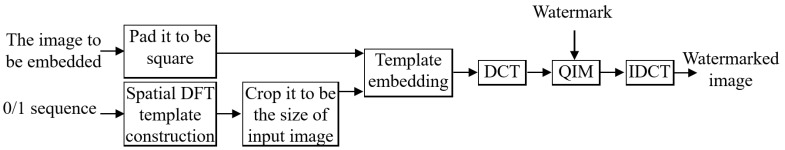
Proposed watermark embedding algorithm.

Figure 5 shows the whole watermark embedding process, and the detailed embedding algorithmic steps are as follows:

Step 0: Generate a spatial template as follows:

Step 0.1. Construct a large-size (e.g., 10,000 × 10,000) square gray-level image *G* with each pixel having the same luminance value 128.

Step 0.2. Apply the DFT to *G* to obtain the DFT spectrum $G_{\mathrm{DFT}}$.

Step 0.3. Design a specific {0,1}-sequence *C* according to the three principles mentioned above, and denote it as a circle.

Step 0.4. Add this circle *C* to the DFT spectrum $G_{\mathrm{DFT}}$ and obtain the modified DFT spectrum $G_{\mathrm{DFT}}^{\prime }$.

Step 0.5. IDFT is applied to the modified DFT spectrum $G_{\mathrm{DFT}}^{\prime }$ to generate the final spatial template *T* to be embedded.

Step 1: Input the cover image *I* to be embedded. If the image size is not square, then it should be padded to be square. Thus, we have the padded image $I_{\mathrm{PAD}}$ to be embedded.

Step 2: Crop the template from the center outward to match the size of the padded image $I_{\mathrm{PAD}}$. Then, the cropped template $T_{\mathrm{CROP}}$ is multiplied by an adaptive coefficient and added to the padded image $I_{\mathrm{PAD}}$ to finish the template-embedding process, obtaining the image $I_{T}$.

Step 3: The image $I_{T}$ is divided into blocks, and DCT is performed on each block.

Step 4: The independent sub-area is settled in the image $I_{T}$ and the encoded watermark information is embedded once. Furthermore, the information header should be embedded in each sub-area to resist the translation attack. A pair of DCT coefficients of each block is chosen, and then QIM is used to modify the difference between the two coefficients to finish embedding the watermark information.

Step 5: Apply the IDCT to all modified DCT blocks to obtain the final watermarked image $I_{W}$, where the padded part should be removed.

#### 2.3.2. Proposed Extraction Algorithm

Our core extraction algorithm also includes two stages. DFT-based extraction is performed first to correct the image, and then DCT-based extraction is performed to extract the information.

(1) DFT-based watermark extraction stage

**Figure 6:**
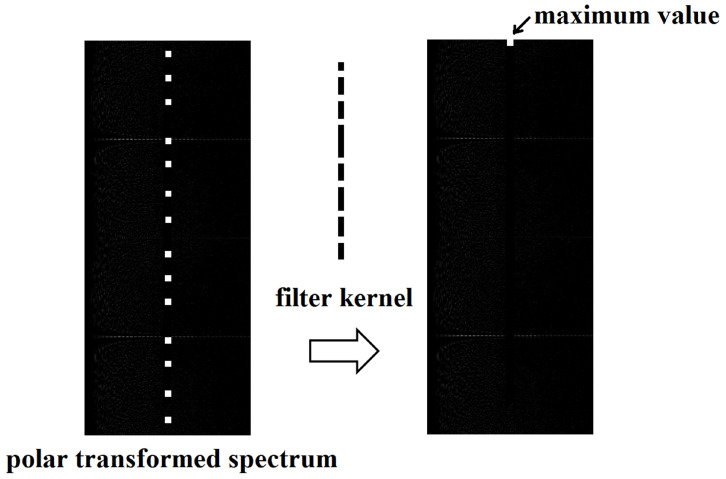
An example of the polar transformed spectrum, constructed filter kernel, and the result of filtering.

As we know, the rotation angle and scaling factor can be found by computing the cross-covariance value, but this process takes too much time. To save the computation time, we design a new method for determining the rotation angle and scale factor. Before detection, the image to be detected can be cropped for faster detection if the image has a large size. Then, DFT is performed on the cropped square image to obtain the spectrum. To reduce possible noises in the DFT spectrum, the spectrum needs to be filtered by the Wiener filter, and then the filtered spectrum needs to be thresholded. If a point value exceeds a certain predefined limit, the point value is divided by the local mean. If the point value does not exceed the limit, the point value is replaced with zero. According to the 0,1-sequence, a filter kernel should be constructed as shown in Figure 6. And the polar transformation needs to be performed on the clean spectrum. Then, the polar transformed spectrum is filtered by the constructed filter kernel, and the maximum value should be found in the filtered map. If the maximum value is larger than the settled threshold, we can say that this image contains the DFT-based watermark; otherwise, there is no DFT-based watermark in the image. And the coordinates of the maximum value are the rotation angle and the circle radius. According to the detected radius, the scaling factor can be computed by:

(3) $$s=\frac{r_{e}}{r_{d}/\left(\mathrm{int}\right)\left(l/2\right)}$$

where $r_{e}$ denotes the circle radius in the embedding process, $r_{d}$ denotes the detected circle radius, and *l* denotes the width of the square image.

(2) DCT-based watermark extraction stage

**Figure 7:**
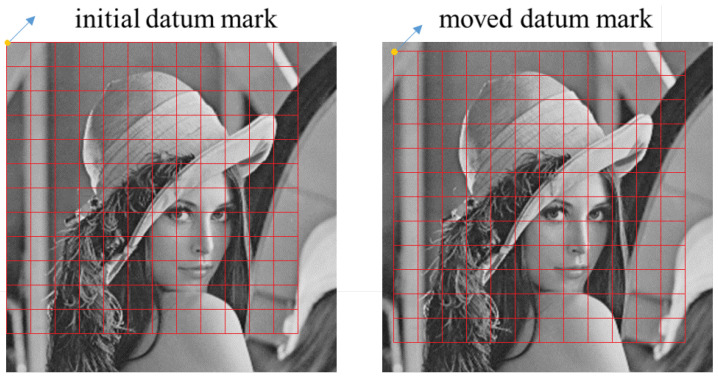
An example of the datum mark for block dividing.

The DCT-based information extraction process is just the inverse process of the DCT-based information embedding process. DCT is performed on each block, and compute the difference between the two selected coefficients, and then QIM is used to extract the watermark bit according to the difference. The information header is searched in the extracted bits, and the position of the sub-area can be determined if an information header is found. If there is no information header in the extracted bits, the position of the datum mark needs to be moved and the blocks need to be divided again, until an information header can be found in the extracted bits. An example is shown in Figure 7. In this method, we use sub-areas and the search of information headers to resist translation and shear attacks, and the differential quantization embedding method has good robustness and imperceptibility.

(3) The whole two-stage watermark extraction process.

**Figure 8:**
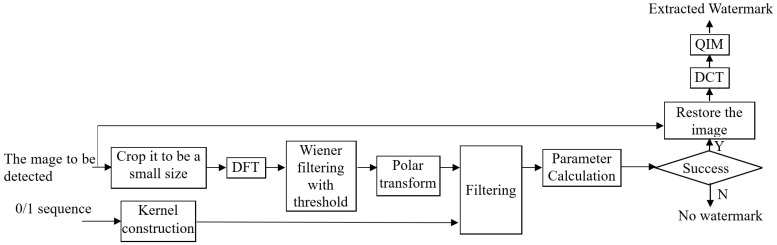
Proposed watermark extraction algorithm.

Figure 8 shows the whole watermark extraction process, and the detailed algorithmic steps are as follows:

Step 1: Input the image to be detected $I^{\prime }$; it can be cropped as $I_{\mathrm{CROP}}^{\prime }$ of size U×U for faster detection if the image has a large size.

Step 2: DFT is applied to the cropped square image $I_{\mathrm{CROP}}^{\prime }$ to obtain its spectrum $I_{\mathrm{DFT}}^{\prime }$, which is filtered by the Wiener filter, and then the filtered spectrum is thresholded, obtaining $I_{\mathrm{DFT}}^{\prime F}$.

Step 3: Polar transform is performed on the clean spectrum to obtain $I_{\mathrm{DFT}}^{\prime \mathrm{FP}}$. Based on the 0,1-sequence, a filter kernel is constructed. Then, the polar transformed spectrum $I_{\mathrm{DFT}}^{\prime \mathrm{FP}}$ is filtered by the constructed filter kernel.

Step 4: Parameter calculation. If the maximum value in the filtered map $I_{\mathrm{DFT}}^{\prime \mathrm{FPF}}$ is less than the threshold, the image contains no DFT-based watermark, and the extraction is terminated. Otherwise, the coordinates of the maximum value are the rotation angle α and the circle radius $r_{d}$. According to the detected radius, $r_{d}$, the scaling factor, *s*, can be computed as the circle radius in the embedding process, $r_{e}$, divided by the detected circle radius, $r_{d}$, and then multiplied by half of the width of the square image, U/2. Then, the input image is restored based on the calculated parameters.

Step 5: The restored image is divided into blocks.

Step 6: Perform DCT on each block, and compute the difference between the two selected coefficients, and then QIM is used to extract the watermark bit according to the difference.

Step 7: The information header is searched in the extracted bits as discussed above. Thus, the final extracted watermark bits can be obtained by removing the header bits.

### 2.4. Proposed Secret Hiding Process

**Figure 9:**
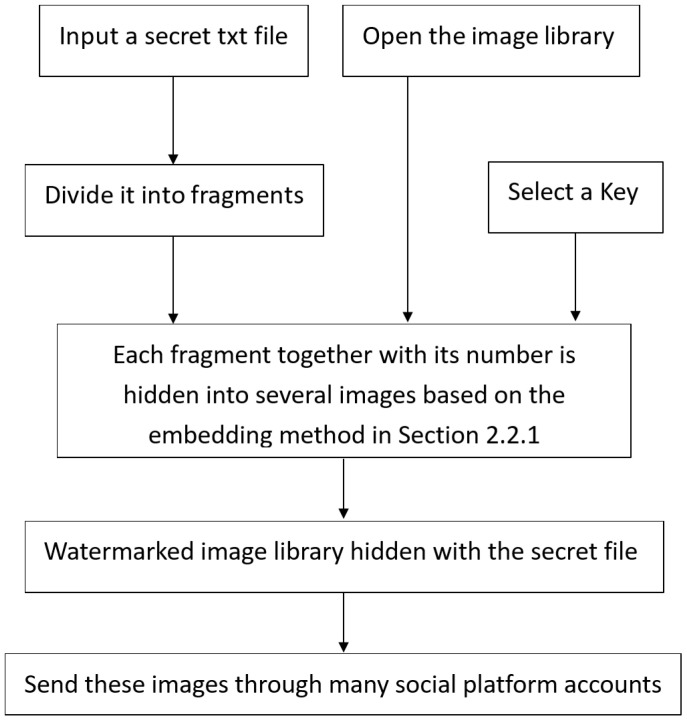
Proposed secret hiding process.

After determining the core watermarking algorithm, we can design the covert communication system by hiding the secret txt file into a large number of images. The secret hiding process is shown in Figure 9. The basic idea is as follows: given an image library, assume there are *N* images in it. Given a text file, assume it contains *M* bytes. Assuming that our robust image watermark embedding algorithm in Section 2.2.1 can embed *K*-bit watermark information in each image, we call it a fragment. Here, *K* is a multiple of 8, and 20 bits of information, including a 10-bit all-zero header and fragment number, must be added to the actual embedding. This means that K+20 bits of information must be embedded in each image. In this way, if a fragment is K/8 bytes, *N* images can hide NK/8 bytes. If M<<NK/8, there is significant redundancy in the embedding space, which means that the maximum redundancy is $r_{\max}=NK/8/M$. If every $r_{\max}$ image is embedded with the same fragment separately, the receiver may recover the complete text file information just once when only $N/r_{\max}$ images are received. If the redundancy is set to *r*, it is obvious that $1\leq r\leq r_{\max}$. Obviously, if NK/8<rM, it is not possible to fully embed all fragments once. The hiding process of the sender in this system can be described as follows:

(1) Step 1: Open the text file to be transmitted confidentially, read the number of bytes *M*, and read all contents in bytes into array *B*. If 8M/K is an integer, let S=8M/K. Otherwise, let S=[8M/K]+1, where [] represents a rounding-down operation.

(2) Step 2: Open the image library and read the number of valid carrier images *N*. Compare the sizes of *N* and rS based on the input redundancy *r*. If N<rS, output a warning stating that the redundancy setting is too large or the number of images is not enough to embed all text fragments redundantly before exiting. If N≥rS, continue.

(3) Step 3: Divide array *B* into *S* fragments. If the last fragment is less than K/8 bytes, add any characters to make up for K/8 bytes. That is $B=\{B_{1},B_{2},...,B_{S}\}$, where $B^{j}=\left\{B_{i}^{j}|i=1,2,...,K/8\right\}$, j=1,2,...,S, and $B_{i}^{j}$ represents the *i*-th byte of the *j*-th fragment.

(4) Step 4: Randomly select P=rS images from N valid images as carrier image set *I*, and then divide these carrier images into *S* groups with *r* images in each group. That is, $I=\{I_{1},I_{2},...,I_{S}\}$, where $I^{j}=\left\{I_{i}^{j}|i=1,2,...,r\right\}$, j=1,2,...,S, $I_{i}^{j}$ represents the *i*-th image of the *j*-th group.

(5) Step 5: Set the fragment number n=1 and the intra-group number m=1.

(6) Step 6: Obtain the *n*th fragment $B^{n}$ from *B*, represent the K/8 bytes of $B^{n}$ as *K*-bit information $B^{n}=\left\{b_{i}^{n}\in \left\{0,1\right\}|i=1,2,...,K\right\}$, and $b_{i}^{n}$ represent the *i*-th bit of the *n*-th fragment. The watermark information to be embedded into the *n*-th set of images is “$w^{n}=0000000000||10-\mathrm{bit}\mathrm{binary}\mathrm{representation}\mathrm{of}\;n||b_{1}^{n}\left|\right|b_{2}^{n}...\left|\right|b_{K}^{n}$”, which means that the 10-bit binary numbers of the fragment number are concatenated first by the 10-bit all zero information header, and then the *K*-bit information is concatenated.

(7) Step 7: Use our robust image watermarking algorithm in Section 2.2.1 to embed K+20-bit information $w^{n}$ in the image, and obtain the image ${\hat{I}}_{m}^{n}$ containing the watermark.

(8) Step 8: m=m+1. If m<r, proceed to Step 7; otherwise, proceed to Step 9.

(9) Step 9: n=n+1. If n<S, proceed to Step 6; otherwise, proceed to Step 10.

(10) Step 10: Collect all P=rS watermarked images $\hat{I}=\left\{{\hat{I}}_{m}^{n}|n=1,2,...,r,m=1,2,...,S\right\}$. Send these watermarked images through various social media platforms.

### 2.5. Proposed Secret Extraction Process

**Figure 10:**
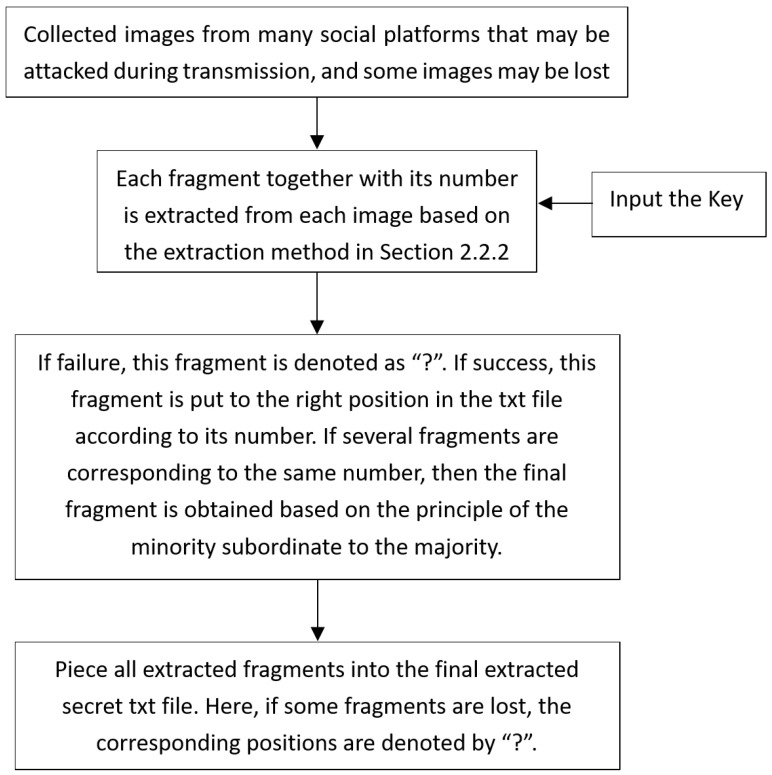
Proposed secret extraction process.

The secret extract process is shown in Figure 10. Assuming that the recipient has obtained *L* images $I^{\prime }=\left\{I_{i}^{\prime }|i=1,2,...,L\right\}$ from various social platforms, usually at least L>S; that is to say, the number of collected images is always greater than the number of fragments *S* of the text file, in order to fully recover the text file. Moreover, some of the collected images may be embedded with the same fragment, or some of the collected images may not be embedded at all. The extraction process of the receiver in this system can be described as follows:

(1) Step 1: Set n=1.

(2) Step 2: Input $I_{n}^{\prime }$, use the strong stick image watermark algorithm in Section 2.2.2 to extract the watermark. If the number of ‘0’s in the first 10 extracted bits is not equal to 10, the extraction fails. Continue to Step 3. Otherwise, continue to extract the hidden fragment numbers and their corresponding *K*-bit fragment information.

(3) Step 3: N=n+1. If n<L, proceed to Step 2; otherwise, proceed to Step 4.

(4) Step 4: According to the fragment number–fragment information combination, the final text file is formed: if multiple fragment data are extracted under a certain fragment number, each bit of fragment information is voted on by a minority following a majority to obtain the final fragment information for that fragment number. If some fragment numbers are missing, all corresponding K/8 byte information will be marked with ‘?’.

## 3. Experimental Results

In order to evaluate the performance of our covert communication system, we conduct experiments in two aspects. First, we evaluate the performance of the proposed core robust image watermarking algorithms. Second, we evaluate the performance of our covert communication scheme.

### 3.1. Performance of Proposed Robust Image Watermarking Method

Here, the performances on effectiveness, robustness, and imperceptibility will be illustrated. The test images come from actual images in the industry, totaling 20,000 images with various resolutions and formats (such as jpg, bmp, and png), and the image size ranges from 700×700 to 2000×2000. We embed an 8×8 binary image (i.e., 64 bits) redundantly in each image.

#### 3.1.1. Efficiency

Firstly, we tested the effectiveness of the proposed scheme. Table 1 presents the statistical results of embedding time and extraction time. From these results, it can be seen that our solution is very fast, all below the second level, fully meeting the real-time requirements. The embedding time ranges from 0.005 s to 0.100 s for images with a size ranging from 700×700 to 2000×2000. The extraction time ranges from 0.005 s to 0.150 s for images with a size ranging from 700×700 to 2000×2000. The reason is that we adopt many techniques to reduce the embedding time and the extraction time. During the embedding process, the DFT calculation is replaced with the spatial method, and the template is prepared in advance, greatly reducing the required embedding time. During the extraction process, the required detection time is reduced by cropping the image before extraction and using the polar transformation and filter. This real-time performance enables our scheme to be used in the application field with online embedding and extraction requirements.

#### 3.1.2. Imperceptibility

**Figure 11:**
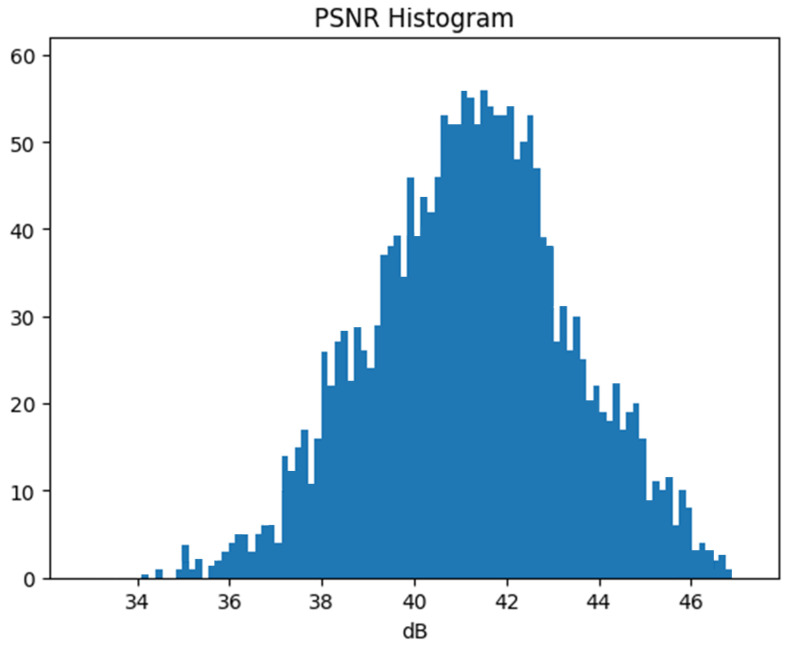
Watermarked PSNR histogram in over 20,000 images.

Next, we test the invisibility of our proposed watermarking scheme. Here, we use PSNR (peak signal-to-noise ratio) as an indicator to evaluate invisibility. The average PSNR of the watermarked image, with 64 bits embedded in over 20,000 images, as shown in Table 1, is 41.05 dB, proving that the proposed scheme has good invisibility. The vivid PSNR histogram is shown in Figure 11, which shows that most of the PSNR values of our scheme fall within the range of 39–43 dB. The reason is that, in the DFT-based embedding stage, our scheme converts the DFT-based transform domain method into a spatial domain-based method with better imperceptibility, while in the DCT-based embedding stage, only two coefficients are modified for each 8×8 block.

#### 3.1.3. Robustness

We also extensively test the robustness of our algorithm against various attacks, including JPEG compression (QF = 10, 30, 50, 70, 90), random cropping (10%, 20%, 30%, 40%, 50% dropped), color degradation (reduced to 16 colors), gamma correction (correction coefficients 0.5, 0.7, 0.9, 1.1, 1.3, 1.5), scaling (scaling coefficients 1/3, 2/3, 1.5, 2), rotation (counterclockwise rotation angles 90, −90, −5, −3, −1, 1, 3, 5 degrees), horizontal flipping, uniform noise (percentages 1%, 5%, 10%, 15%), contrast change (change ratios −0.25, −0.15, 0.15, 0.25), brightness change (change ratios −0.25, −0.15, 0.15, 0.25), median filtering (filter kernel size 3×3). Here, we use accuracy (defined as the ratio of the number of images from which the watermark is completely and correctly extracted out of 20,000) to characterize robustness. Table 2 shows the extraction accuracy for over 20,000 images against the above attacks, most of which are above 95%, indicating that the proposed scheme has strong robustness. The reasons for high robustness lie in two aspects: (1) We use a multi-domain watermarking scheme with two layers, where the DFT-based watermarking layer is proposed for synchronization correction and the DCT-based watermarking layer is proposed for resisting translation attacks. (2) We combine several techniques together, such as synchronization correction, information headers, and error-correcting codes, during both embedding and extraction processes to make our scheme fast enough as well as robust to most attacks that might occur in real applications.

#### 3.1.4. Comparison with Existing Algorithms

Finally, we compare the robustness of our proposed approach with some state-of-the-art methods. Our scheme can deal with multiple attacks and multiple joint attacks. The types of attacks used for algorithm comparison include rotation, scaling, translation, JPEG compression, cropping, collage, gamma correction, white noise, contrast adjustment, brightness adjustment, and median filtering. We use accuracy, which is defined as the ratio of the number of images from which the watermark is completely and correctly extracted out of 20,000, to characterize robustness. The comparison results are shown in Table 3, and it can be found that our proposed scheme has better robustness than other schemes (e.g., the fractional exponent moment-based method [20], SVD-based contourlet transformation method [38], DWT-DCT combination method [25], spatial domain-based DFT method [42], synchronous correction method [39]), as well as the DHT (discrete Hartley transform)-based specific-component quantization method [37]. We also compare the robust image steganography method proposed in [11].

From Table 3, we can see that our scheme has the best JPEG, translation, rotation, collage, and cropping robustness; the reason is that our method has two watermarking layers that are combined tightly to deal with all possible kinds of attacks, proving particularly effective for these attacks. Among existing schemes, the performance of the robust watermarking scheme [37,38,39] is close to that of our scheme. The method in [11] has the overall worst performance. In fact, the robust image steganography method proposed in [11] is designed for JPEG attacks only. All algorithms have good JPEG and white-noise robustness. All robust image watermarking methods have good median-filtering robustness. All methods have similar contrast, luminance, and Gamma-correction robustness. Our scheme, together with [37,38,39], has good rotation, cropping, translation, and collage robustness, but [11,25,38,42] has relatively worse rotation, cropping, translation, collage robustness. The image steganography schemes generally pay more attention to capacity and invisibility. The robustness performance is not that good, while the image watermarking schemes pay more attention to robustness; thus, their capacity is not high.

### 3.2. Performance of Proposed Covert Communication Scheme

**Figure 12:**
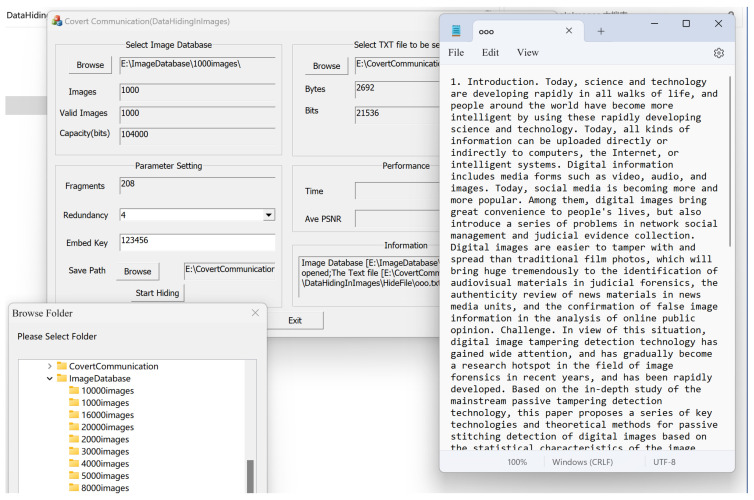
Demonstration interface for the sender’s hiding process.

In order to demonstrate the effectiveness of fragmented hidden communication in this article, we specially wrote a program interface to simulate the entire process. The demonstration interface of the sender’s hidden process is shown in Figure 12. The main idea is to fragment the secret information to be transmitted, and then use the robust digital image steganography algorithm in Section 2.2 to redundantly embed it into a number of images and extract the secret information based on the received images. We first provide an example to show the hiding and extraction processes. Then, we test the performance of our covert communication scheme under different parameters.

#### 3.2.1. An Implementation Example

**Figure 13:**
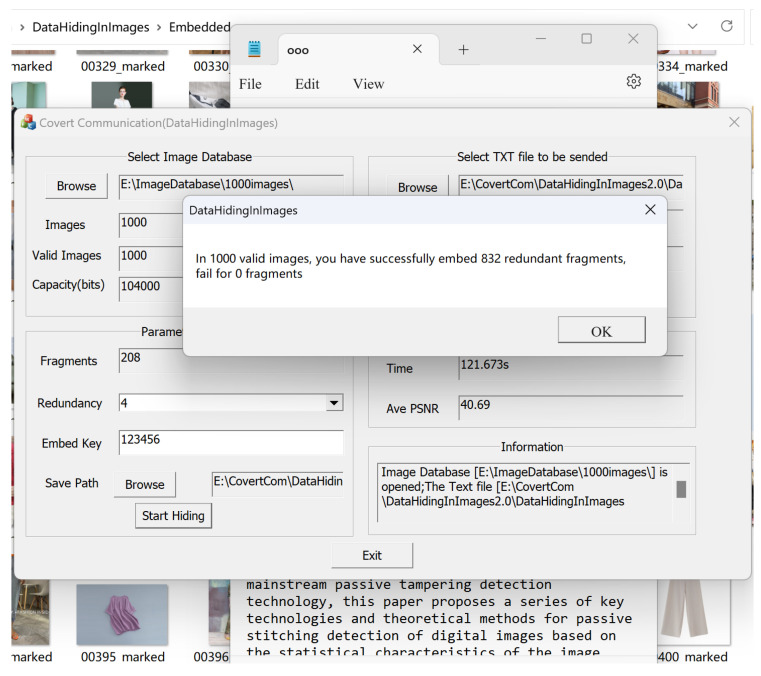
Hiding result for the sender’s hiding process.

Figure 12 shows an example of transmitting the secret information i.e., the text file named ooo.txt, which is divided into 208 fragments, and then we use the robust embedding algorithm in Section 2.2.2 to redundantly embed it into 1000 images. The redundancy is set to ‘4’, indicating that each fragment is embedded into 4 images. When we click to start hiding, we obtain the running result interface, as shown in Figure 13, with a total of 832 fragments embedded (208×4=832). It can be seen that the embedding time is 121 s, and the average PSNR is 40.69 dB. The 832 watermarked images and 168 non-watermarked images are stored in the designated folder.

**Figure 14:**
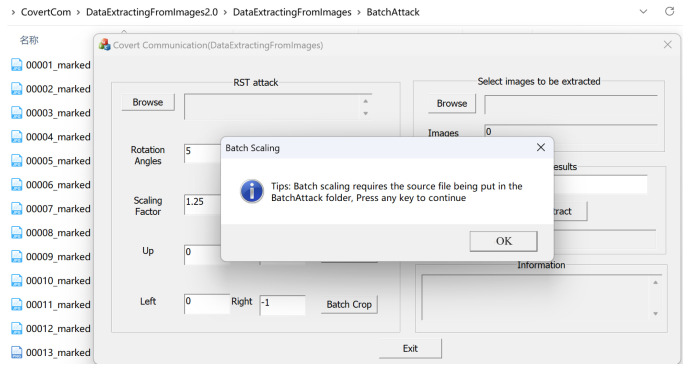
Simulate batch scaling attacks.

**Figure 15:**
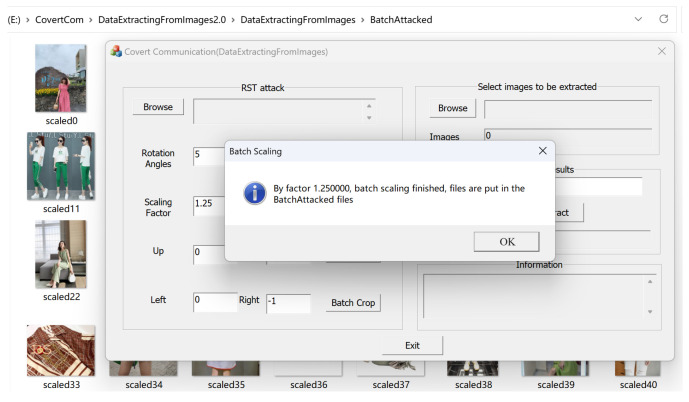
Batch scaling results.

The demonstration interface for simulating the attack process is shown in Figure 14. We simulate placing the 1000 watermarked images in the designated BatchAttack folder and suffer a scaling attack of 1.25. After clicking on confirm, we performed batch scaling, as shown in Figure 15. The results are stored in the BatchAttached folder.

**Figure 16:**
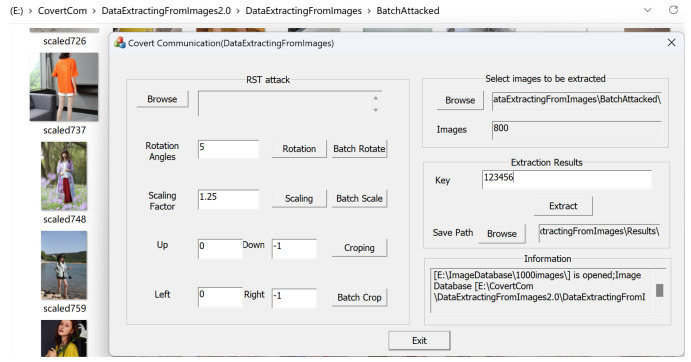
The extraction process.

**Figure 17:**
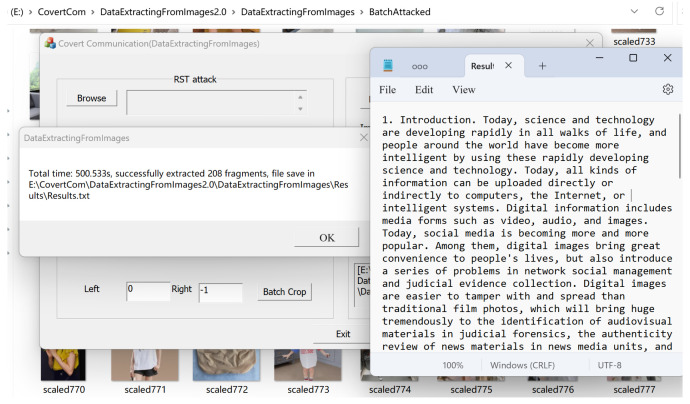
The extraction results.

The demonstration interface of the recipient’s extraction process is shown in Figure 16. We open the BatchAttached directory (originally 1000 images) and randomly delete 200 scaled watermarked images from that directory, leaving 800 images remaining. We click to extract and obtain the results shown in Figure 17, which takes 500 s. The extracted text file shown in Figure 17 indicates that even after experiencing a 1.25 scaling attack and deleting 200 images, the original text file can be completely restored. In most cases, using our covert communication system, the vast majority of the text file content can be recovered from the extracted fragments. If all hidden fragmented images can be obtained, the original text file can be completely restored.

#### 3.2.2. Performance Test

Next, we test the performance of the proposed covert communication scheme. First, we test the performance without any attack. Then, we test the performance under the loss of a certain percentage of images. Finally, we test the performance under different attacks.

To evaluate the performance without any attack, we use several databases of different sizes, i.e., the image databases with 1000 images, 2000 images, 4000 images, 8000 images, and 16,000 images, respectively. The secret txt file is also ooo.txt, which has 2692 bytes. The images in these databases are randomly selected from the large database with 20,000 images. The performance indicators include the average PSNR, hiding time $t_{h}$, extraction time $t_{e}$, and success rate *s* (which is defined as the successfully restored bytes divided by the whole bytes in the txt files). The test results are shown in Table 4, where *r* denotes the redundancy (i.e., the number of images that are embedded with the same fragment), *N* denotes the number of images in a database, $N_{h}$ denotes the number of fragments hidden, and $N_{u}$ denotes the number of non-watermarked images. Obviously, we have $N=N_{h}+N_{u}$. From Table 4, we can see that both the embedding time and extraction time are nearly proportional to the number of images, our scheme has good imperceptibility, and the receiver can restore the secret file completely under no attack.

To evaluate performance under the loss of a certain percentage of images, we use a database with 1000 images. We set r=4, meaning the number of images embedded with the same fragment is 4. Since there are 208 fragments, the number of images embedded with fragments is $N_{h}=832=208\times 4$, and the number of images not embedded with any fragment is $N_{u}=1000-832=168$. At the receiver, we randomly remove 10%, 20%, 30%, 40%, 50%, 60%, 70% and 80% of the received images. Their corresponding extraction time, $t_{e}$, and success rate, *s* are, shown in Table 5. We can see that our scheme can successfully restore the secret file completely even if 30% of the received images are lost. When 80% of received images are lost, that is, based on the remaining 200 images, our scheme can still restore 61.1% of the secret file.

To evaluate the performance under different RST attacks, we also use the database with 1000 images. We set r=4, $N_{h}=832$, and $N_{u}=168$. Before extraction, the received images suffer from rotation, scaling, and cropping attacks. The rotation angles are 1° and 5°, respectively, the scaling factors are 1.25 and 0.8 respectively, and the dropped percentages of the cropping attack are 10% and 25%, respectively. Table 6 shows the success rates *s* under different attacks. We can see that our scheme can successfully restore the secret file completely under different RST attacks.

To evaluate the performance under different combined attacks, we also use the database with 1000 images. We set r=4, $N_{h}=832$, and $N_{u}=168$. Before extraction, the received images suffer from rotation+scaling, scaling+cropping, JPEG (QF = 80) + cropping, white noise (1%)+scaling, median filtering (3×3) + rotation attacks. Here, the rotation angle is 1°, the scaling factor is 1.25, and the dropped percentage of the cropping attack is 5%. Table 7 shows the success rates *s* under different combined attacks. We can see that our scheme can successfully restore the secret file completely under different combined attacks.

Finally, we should point out that our current scheme has a relatively low efficiency since a text file should be hidden in a large image database; thus, the hiding capacity per image is very limited. However, the aim of this paper is to demonstrate our idea of hiding secrets in big data, with the main focus on the secrecy of the transmission system.

## 4. Conclusions

This article focuses on robust digital image watermarking algorithms in the field of secure communication. We consider the situation where we have large image databases at hand and need to transmit a secret file over social platforms susceptible to attacks. Under the big data situation, capacity and security are not as important for a single image, while robustness against attacks during transmission is very important. Thus, it is proposed that robust image watermarking technology can be fully utilized to address the problem in noisy channels under big data environments. Robust watermarking technology aims to accurately detect watermark information even when watermarked images are subjected to various attacks and processing. To make the watermarking scheme suitable for secure communication in lossy channels, we designed a robust digital image watermarking method. The innovation and characteristics of this scheme can be summarized as follows: Firstly, a DFT-based watermarking stage for synchronization is proposed, which has high embedding and detection efficiency and good invisibility. Secondly, a DCT-based watermarking stage is proposed, which can resist translation attacks, JPEG attacks, and other common attacks. Thirdly, the watermarking scheme has the characteristics of high efficiency, good robustness, and good invisibility. Then, we cleverly designed a fragmented secure communication application system, which achieved good application results based on the core algorithm mentioned above. Experiments have shown that the scheme of fragmenting and hiding redundant transmission content into a large number of images is effective and practical. Even after all image carriers undergo geometric or hybrid attacks, based on partially received images, the vast majority of transmission content can still be recovered from the extracted fragments. If all watermarked images can be obtained, the original text file can be completely restored. However, the limitations of our current scheme lie in two aspects: (1) Our framework has low efficiency, and a text file should be hidden in a large amount of carrier data; thus, the hiding capacity is limited. (2) Our watermarking scheme is not very robust to contrast and luminance changes. Our future work will concentrate on improving the hiding capacity per image and the robustness to contrast and luminance changes.

## Figures and Tables

**Table 1:** The efficiency of embedding and extracting watermark algorithms.

Machine performance	i7-9750H, CPU:2.60 GHz, RAM:16.0 GB
800×800 image embedding time	0.019 s
1500×2000 image embedding time	0.098 s
Average PSNR	41.05 dB
800×800 image extraction time	0.069 s
1500×2000 image extraction time	0.115 s

**Table 2:** The extraction success rate of the proposed watermarking algorithm under various attacks.

Attacks	Para- Meter	Success Rate	Attacks	Para- Meter	Success Rate
None	-	97.40%	Rotation|| Angles	90	97.80%
JPEG ||QF	10	30.50%		−90	97.90%
	30	84.70%		−5	97.80%
	50	94.10%		−3	95.90%
	70	97.30%		−1	96.40%
	90	97.60%		1	96.80%
Random cutting ||Percentage	10%	76.30%		3	96.70%
	20%	89.20%		5	97.10%
	30%	90.60%	Flip horizontally||-	-	97.30%
	40%	95.80%	Uniform noise ||Ratio	1%	89.60%
	50%	97.10%		5%	72.40%
Color degradation ||Colors	16	95.10%		10%	58.00%
Gamma Correction ||Coefficient	0.5	96.00%		15%	50.30%
	0.7	96.80%	Contrast change ||Ratio	−0.25	98.00%
	0.9	97.80%		−0.15	98.00%
	1.1	98.10%		0.25	94.30%
	1.3	97.90%		0.15	94.80%
	1.5	97.90%	Luminance change ||Ratio	−0.25	97.30%
Scaling ||Factor	1/3	100.00%		−0.15	97.70%
	2/3	92.00%		0.15	94.00%
	1.5	95.90%		0.25	91.10%
	2	93.70%	Median filtering ||Kernel size	3	92.90%

**Table 3:** Comparison of extraction accuracy for over 20,000 test images.

Method	Our	[20]	[38]	[25]	[42]	[39]	[37]	[11]
Year	-	2021	2021	2022	2022	2018	2023	2023
JPEG-50	0.976	0.973	0.969	0.959	0.971	0.915	0.948	0.968
Rotate-5°	0.983	0.982	0.325	0.175	0.245	0.980	0.978	0.125
Crop-50%	0.994	0.991	0.735	0.850	0.875	0.923	0.989	0.110
Gamma-0.5	0.886	0.843	0.934	0.845	0.812	0.853	0.832	0.789
Gamma-1.5	0.950	0.875	0.943	0.952	0.886	0.885	0.901	0.812
Scaling-0.6	0.958	0.948	0.946	0.792	0.946	0.966	0.943	0.015
Scaling-1.5	0.949	0.952	0.939	0.940	0.950	0.948	0.932	0.010
Translation	1.0	0.990	0.125	0.125	0.234	1.0	0.910	0.100
Collage	1.0	0.924	0.245	0.245	0.158	0.986	0.925	0.050
White-noise (20 dB)	0.988	0.994	0.923	0.970	0.958	0.989	0.978	0.876
Contrast-50%	0.540	0.498	0.515	0.532	0.486	0.581	0.712	0.564
Luminance-50%	0.768	0.675	0.698	0.724	0.751	0.743	0.815	0.712
Median-3 × 3	0.965	0.964	0.910	0.945	0.925	0.956	0.943	0.678

**Table 4:** The performance of our scheme without any attack.

*N*	*r*	$N_{h}$	$N_{u}$	PSNR (dB)	$t_{h}$ (s)	$t_{e}$ (s)	*s*
1000	4	832	168	40.69	121.7	500.5	100%
2000	9	1872	128	40.52	252.6	1004.1	100%
4000	19	3952	48	40.72	489.3	2015.4	100%
8000	38	7904	96	40.58	970.1	4003.7	100%
16,000	76	15,808	192	40.59	1942.5	8011.9	100%

**Table 5:** The performance of our scheme under the loss of a certain percentage of images.

Percentage	10%	20%	30%	40%	50%	60%	70%	80%
$t_{e}$ (s)	381.1	309.5	299.5	244.9	177.8	121.7	113.7	53.5
*s*	100%	100%	100%	97.6%	95.2%	89.4%	76.4%	61.1%

**Table 6:** The performance of our scheme under different RST attacks.

Attacks	Rotation by 1°	Rotation by 5°	Scaling 1.25	Scaling 0.8	Cropping 10%	Cropping 25%
$t_{e}$(s)	438.6	420.9	558.9	231.7	225.6	215.7
*s*	100%	100%	100%	100%	100%	100%

**Table 7:** The performance of our scheme under different combined attacks.

Attacks	Rotation +Scaling	Scaling +Cropping	JPEG (QF = 80) +Cropping	White Noise (1%) +Scaling	Median Filtering (3×3) + Rotation
$t_{e}$(s)	528.6	515.7	498.7	520.1	531.6
*s*	100%	100%	100%	100%	100%

## Data Availability

Data are contained within the article.
